# Growth Parameters and Growth-Related Hormone Profile in a Herd of Cattle up to 4 Years of Age Derived from Assisted Reproductive Technologies

**DOI:** 10.3390/ani15050631

**Published:** 2025-02-21

**Authors:** Sonia Heras, Jordana Sena Lopes, Armando Quintero-Moreno, Jon Romero-Aguirregomezcorta, Sebastian Canovas, Raquel Romar, Pilar Coy

**Affiliations:** Physiology of Reproduction Group, Department of Physiology, IMIB-Pascual Parrilla, University of Murcia, 30100 Murcia, Spain; sonia.heras@um.es (S.H.); jordana.lopes@uevora.pt (J.S.L.); armando.quintero@um.es (A.Q.-M.); jon.romero@um.es (J.R.-A.); scber@um.es (S.C.); rromar@um.es (R.R.)

**Keywords:** animal health, assisted reproductive technologies, cattle, growth parameters, growth-related hormones, long-term impact

## Abstract

Assisted reproductive technologies (ART) are routinely used in livestock to generate animals of high genetic value. Despite representing an outstanding accomplishment, recent studies suggest differences in health, fertility, and gestational length of in vitro-produced compared to in vivo-derived animals. Currently, there are no data available on the long-term effects of ART on growth and development. This observational study aimed to understand the relationship between growth and growth-influencing hormones in a herd of cattle derived from artificial insemination (AI) or from in vitro-produced embryos either with BSA (C-IVP) or with reproductive fluids (RF-IVP) as a protein source in culture. Cortisol was associated positively with weight in AI and negatively with body length in males. Thyroxine decreased with age, and it was positively associated with thoracic circumference in RF-IVP. Insulin-like growth factor-1 was greater in RF-IVP than in C-IVP, and it was positively associated with body length and withers height. Growth hormone was greater in females than in males and RF-IVP compared to AI and C-IVP. In conclusion, we present here the first datasets on growth parameters and growth-influencing hormones in cattle from birth to 4 years of age without observing major evidence of differences depending on the embryo origin.

## Simple Summary

The use of assisted reproductive technologies is common in livestock to reduce animal transportation and generational intervals and to produce animals of high genetic value. However, these technologies might have an impact on health, milk production, fertility, and gestational length, with no data currently available on the effects on growth. Our aim was to study the relationship between growth parameters and growth and metabolism influencing hormones from birth up to four years of age in cattle produced by different assisted reproductive technologies. Despite the small differences observed between the groups in some of the parameters studied, we did not observe any differences of significance in development between the animals produced by different assisted reproductive technologies. Nevertheless, further studies with a larger number of animals are needed to confirm these observations. Additionally, this study contributes with novel data on growth-influencing hormone concentrations in cattle throughout life.

## 1. Introduction

Developments in assisted reproductive technologies (ART) have significantly reshaped the livestock production landscape, offering novel approaches to enhance breeding efficiency and genetic progress. Among the major ART contributing to the accelerated genetic improvement of cattle populations are artificial insemination (AI) and in vitro embryo production, followed by embryo transfer (IVP-ET) [1]. Artificial insemination has been widely used in the cattle industry for decades. It is accepted that there are no major differences between AI-derived and naturally conceived animals. However, this may not be the case when compared to IVP-ET animals [2]. The number of IVP-ET transfers has been increasing yearly, with more than 1 million reported by the International Embryo Technology Society (IETS) in 2022 [3]. Information on the phenotypical traits of these animals in adulthood is limited. Recent studies suggest differences in health, milk production, fertility, and gestational length compared to AI or multiple ovulation-embryo transfer-derived animals [2,4]. However, information on other traits related to growth and its endocrine regulation from birth to adulthood is scarce. In the field of livestock management, it is essential to improve our understanding of the physiological mechanisms that control the growth and development of animals based on their origin. This will enable us to determine whether the performance of IVP-ET animals is comparable to that of their in vivo-derived counterparts, thereby contributing to the improvement of the industry and the welfare of the animals. Indeed, growth parameters and hormone concentrations play pivotal roles in shaping the overall productivity and well-being of cattle. Among the key hormones influencing growth and metabolism, growth hormone (GH), insulin-like growth factor-1 (IGF-1), thyroxine (T4), and cortisol stand out as central regulators [5]. The intricate interplay between these hormones is known to regulate various physiological processes, including cell proliferation, protein synthesis, and metabolic homeostasis [6,7]. Besides their role in the regulation of metabolism, the measurement of cortisol levels is broadly used as a biomarker of animal’s stress levels and, thus, their well-being [8].

A study exploring the metabolic, hematological, and endocrine status of IVP-ET calves and their implications in growth performance and feed efficiency until 16 weeks of age highlighted the need for a longer-term investigation [9]. In a previous year-long study by our group [10], the growth, hematological, and biochemical parameters of a small herd of calves derived from IVP-ET and AI were compared. We found that all the analyzed variables were within the physiological range and very similar between AI and IVP-ET calves. However, considering the life expectancy of cattle, a longer-term investigation was still necessary.

Therefore, in the present observational study, we aim to further analyze the growth parameters of the same animals from the previous study [10] until 4 years of age and to include the hormones that could influence growth and report animal welfare (GH, IGF-1, T4, and cortisol). This animal herd offers a unique opportunity to provide baseline values for the hormones governing growth in IVP-ET and AI-derived male and female cattle over time. We hypothesized that IVP-derived animals could perform similarly to AI-derived animals in terms of growth and growth regulation, providing a safe method to accelerate genetic improvement in the cattle industry.

## 2. Material and Methods

### 2.1. Animals

The 19 animals included in this study were generated for a previous study [11]. Animals from the AI group were produced by artificial insemination with frozen–thawed semen. Multiparous Holstein cows were synchronized with double-Ovsynch protocol [12] and inseminated 16 to 20 h after the last GnRH injection (day 0, presumptive day of estrus).

For the in vitro groups (C-IVP and RF-IVP), embryos were produced using slaughterhouse-obtained ovaries (crossbred Charolais and/or Limousin), as described by Lopes et al. [11]. Briefly, groups of 50 cumulus-oocytes complexes (COCs) were matured in a maturation medium consisting of TCM199 supplemented with 10% fetal calf serum and 10 ng/mL epidermal growth factor for 24 h at 38.5 °C in 5% CO_2_-in-air. Frozen–thawed semen was passed through a discontinuous Bovipure gradient (40 and 80% (*v*/*v*); Nidacon, Sweeden). Matured oocytes were incubated with 1 × 10^6^ spermatozoa/mL in fertilization medium (Tyrode’s medium with 25 mM bicarbonate, 22 mM sodium lactate, 1 mM sodium pyruvate, 6 mg/mL fatty acid-free BSA and 10 mg/mL heparin sodium salt) for 18–20 h at 38.5 °C in 5% CO_2_-in-air. Presumptive zygotes were vortexed to remove the remaining cumulus cells and spermatozoa and, for each replicate, were divided into two groups depending on the culture medium (SOF) supplementation. For the C-IVP group, SOF was supplemented with 3 mg/mL of BSA from day 1 to day 8, and the medium was refreshed at day 4. For the RF-IVP group, SOF was supplemented with 1.25% (*v*/*v*) of oviductal fluid (from early luteal phase, NaturARTs BOF-EL, Embryocloud, Spain) from day 1 to day 4, and with 1.25% (*v*/*v*) of uterine fluid (from mid-luteal phase, NaturARTs BUF-ML, Embryocloud, Spain) from day 4 to day 8. Presumptive zygotes were cultured in groups of 25 per 25 µL drops covered with paraffin oil (Nidoil, Nidacon, Sweeden) at 38.5 °C in 5% CO_2_ and 5% O_2_. Blastocysts were vitrified on their days 7 or 8 and then transferred to Holstein multiparous cows (synchronized with double-Ovsynch protocol [12]) from the same commercial farm as the AI group (El Barranquillo SL, Torre Pacheco, Murcia, Spain). Recipient cows were evaluated with ultrasound the day prior to transfer. Recipients were on their day 6, 7, or 8 of the estrus cycle, and embryo transfer (one embryo per recipient) was made non-surgically to the ipsilateral uterine horn from ovulation [11]. All the animals were sired by the same bull (*Asturiana de los Valles* breed) and were born in the span of nine months (August 2018–May 2019) (Supplementary Table S1).

The recipients and the artificially inseminated animals were housed in 2000 square meter open-air yards with roofs to protect them from rain and sun in Murcia, Spain, a geographical area where the average temperature is above 10 °C from December to March and between 26 °C and 29 °C from June to September. Calves from all the experimental groups were separated from their mothers immediately after birth and housed in separate 1.2 square meter blocks that were roofed but open on one side.

Colostrum (prepared from a pool of colostrum of different cows) was administered to the calves twice daily for the first three days of life, starting with 3 L in the first 2 h after birth and repeating every 12 h. Following this, they were fed 3 L of milk replacer (Sprayfo Royal 60, 140 g/L of water, Trouw Nutrition, Sloten, Netherlands) twice daily. At around six days old, the calves were transported to the Veterinary Farm at the University of Murcia, Spain, where they continued the same feeding regimen, increasing to 4 L twice daily until day 45 of age. Fresh water was available ad libitum. Here, the calves were housed in small individual pens of 3 square meters, side by side, in a covered enclosure with one side free of walls. At 15 days of age, the animals were grouped together in the same outdoor pen with access to both calf starter feed and forage. The milk replacer frequency was reduced to once daily at 45 days of age and completely discontinued at 60 days (weaning age). The calves continued to receive the calf starter ad libitum until three months of age. From age three to six months (adaptation phase), the diet consisted of a grain mix and hay, transitioning at six months (growth phase) to a diet of concentrate mixed with straw. At this age, the animals were regrouped by sex in larger facilities, similar to those in the commercial farm. At 12 months of age, a maintenance concentrate was gradually introduced to the animals’ diet (maintenance phase). Fresh water and straw were provided ad libitum throughout.

During the adaptation phase, each animal consumed approximately 6 kg/day of concentrate. Chemical composition: crude protein (13.1%), energy digestible intestinal protein (PDIE; 9.9%), crude fiber (6.7%), starch (39.0%), crude fat (4.5%), feed units for cattle (UFC; 101.1). Ingredients: barley, corn, corn DGGS, gluten feed, soy hull, calcium carbonate, stabilized fat, antacid buffer, salt, and a mineral and vitamin premix with yeast.

During the growth phase, animals consumed approximately 8 kg/day of growth feed until approximately 12 months of age. Chemical composition: crude protein (13.8%), PDIE (10.4%), crude fiber (4.1%), starch (42.2%), crude fat (5.8%), UFC (107.3). Ingredients: corn, barley, corn DGGS, gluten feed, soy hulls, stabilized fat, calcium carbonate, antacid buffer, salt, mineral and vitamin premix with yeast, and urea.

At the maintenance phase (from 12 months to the end of this study), animals consumed approximately 10 kg/day of maintenance concentrate. The chemical composition of the concentrate was as follows: crude protein (15.5%), crude fiber (9.3%), crude ash (7.9%), crude fat (2.1%), calcium (1.40%), phosphorus (0.51%), and sodium (0.41%). The concentrate ingredients included wheat bran, wheat, soybean hulls, corn rootlet, calcium carbonate, sugar beet molasses, sunflower seed extraction meal, sodium chloride, barley, diatomaceous earth, mineral and vitamin premix, and urea.

### 2.2. Experimental Design

**Figure 1:**
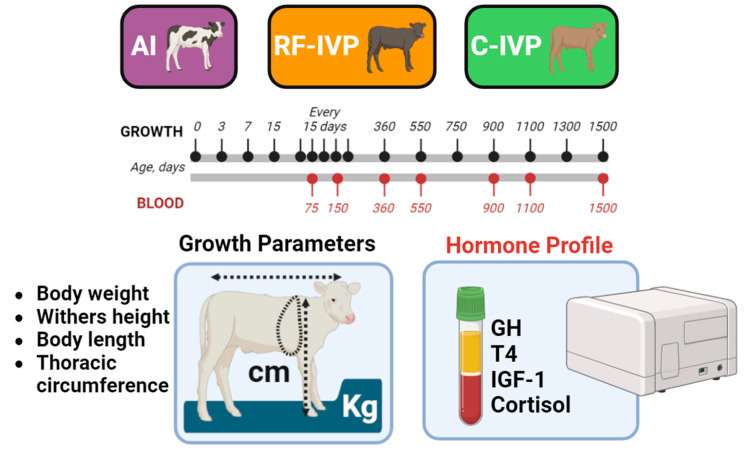
Experimental design. The hormone profile (GH, T4, IGF-1, and cortisol) and the growth parameters (body weight, withers height, body length, and thoracic circumference) in 19 male and female cattle produced by different reproductive technologies (AI: artificial insemination; RF-IVP: reproductive fluids-in vitro production; C-IVP: control-in vitro production), from birth to 1500 days of age, were analyzed.

In this study, we analyzed the hormone profile (GH, IGF-1, T4, and cortisol), the growth parameters (body weight, withers height, thoracic circumference, and body length), and their interactions in 19 male and female cattle produced by different reproductive technologies, from birth to 1500 days of age (Figure 1).

Of those animals, seven (five males and two females) were derived from artificial insemination (AI group), and the rest by in vitro embryo transfer. Five of them (four males and one female) were produced by adding reproductive fluids (oviductal fluid from day 1 to day 4 and uterine fluid from day 4 to day 8) into the culture medium (RF-IVP group), and seven (four males and three females) using BSA from day 1 to day 8 as the only protein source in the culture medium (C-IVP group).

As this was an observational study on an extant herd of animals, and there were no options to design the experimental groups in an appropriate way, the primary focus of this study was not to determine the potential differences among the groups. Rather, the objective was to compile reference values of the hormones and growth parameters analyzed, to gain insight into the evolution of the parameters studied throughout life, and to explore the potential disparities between sexes.

### 2.3. Physical Parameters

Physical examination of the animals took place at birth (0; before colostrum intake) and approximately at 3, 7, 15, every 15 days until day 360, 550, 750, 900, 1100, 1300, and 1500 days of age. The real date of the measurement was used for this analysis. The following parameters were studied: body weight (kg) measured with a weighing scale (Baxtran BR 15), body length (cm) as the distance from the crown of the head to the base of the tail, height at withers (cm), and thoracic circumference (cm) measured at the widest point at withers, all measured with a zoometric measuring tape (Kamer, Ukal).

Data on the physical parameters of the animals from birth to 360 days of age have already been included in a previous study [10]. However, that information was essential in the present study in order to evaluate the interaction between growth-related hormones and growth parameters during the exponential growth phase.

### 2.4. Blood Collection

Blood samples were collected from the jugular vein (calves < 6 months of age) or the caudal vein (animals > 6 months of age) using lithium heparin vacutainer tubes (BD Vacutainer, BD, Madrid, Spain), at approximately 75, 150, 360, 550, 900, 1100, and 1500 days of age. Blood sample collection happened between 8 and 11 am. After collection, blood was centrifuged at 1000× *g* for 15 min, and plasma was stored at −80 °C for analysis.

### 2.5. Hormone Measurements

Plasma hormone concentrations (IGF-1 ref. LKIGF1; Cortisol ref. LKCO1; T4 ref. LKCT1) were determined using a solid-phase, enzyme-labeled competitive chemiluminescent enzyme immunoassay (Immulite 1000; Siemens Healthineers, Los Angeles, CA, USA), according to the manufacturer’s instructions. The concentration of GH was measured by an ELISA competitive assay (CSB-E13443B; Cusabio Biotech Co., Wuhan, China). The optical density was determined by an Epoch 2 microplate spectrophotometer (Agilent BioTek, Santa Clara, CA, USA). All assays showed intra and interassay imprecision lower than 15% and were linear after serial sample dilutions. Detection limits were 14.4 ng/mL for IGF-1, 0.05 ug/dL for Cortisol, 0.12 ug/dL for T4, and 1.25 ng/mL for GH. All measurements fell within the detection range of each assay, except for the GH determination of 13 samples being over the upper detection limit. Those measurements were excluded from this analysis.

### 2.6. Statistical Analysis

Data were analyzed using a linear mixed-effect model, with the predictive variables (group, hormone, sex, and age) as fixed effects and the ID of each animal as a random effect to account for the repeated measurements. A logarithmic adjustment was used between age and growth parameters. For the study of the interaction between the hormones and the growth parameters, interaction terms were included in the model for each combination of interest. The likelihood-ratio test was performed to calculate the significant level of the predictive variables and the interactions. Benjamini-Hochberg procedure was used for *p*-value correction after multiple comparisons to control the False Discovery Rate. The statistical software used for this analysis was R version 4.3.1. R Core Team (2023). Data were considered significant when *p* < 0.05, and a tendency was considered when *p* = 0.06.

## 3. Results

The average values of hormone levels and growth parameters per experimental group and age are available in Supplementary Tables S2 and S3, respectively.

### 3.1. Growth Parameters

**Figure 2:**
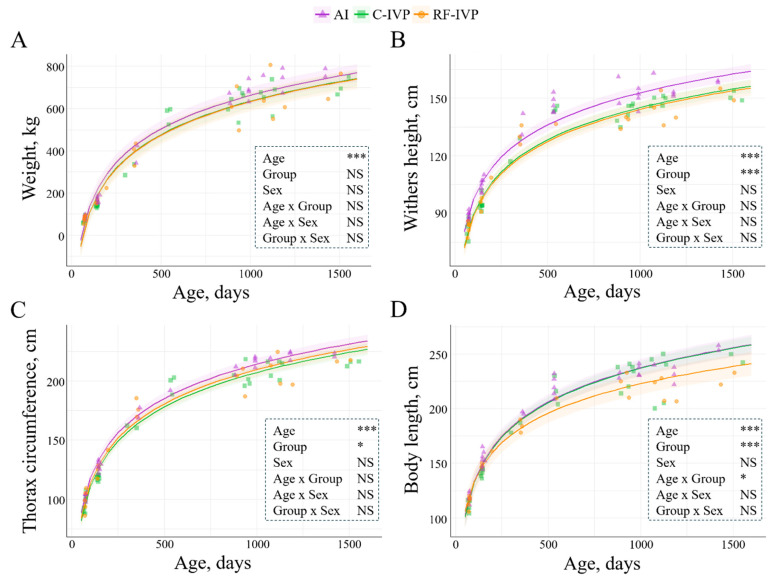
Evolution of the growth parameters included in the study considering age, group, sex, and their interactions. The lines represent the mean values of the growth parameters, and the haloes represent the 95% confidence interval. (**A**) Weight significantly increased with age (*p* < 2.0 × 10^−16^). (**B**) Withers height increased with age (*p* < 2.2 × 10^−16^) and was significantly greater in AI compared to the in vitro counterparts (*p* = 0.0008). (**C**) Thorax circumference increased with age (*p* < 2.0 × 10^−16^), and it was greater in AI compared to C-IVP (*p* = 0.044). (**D**) Body length increased with age (*p* < 2.2 × 10^−16^), and it was greater in AI and C-IVP compared to RF-IVP (*p* = 5.6 × 10^−6^). Only variables with significant differences were represented. Signification codes: NS, not significant; * < 0.05; *** < 0.001.

All growth parameters increased with age. Weight was not influenced by the experimental group or sex; it was only influenced by age (Figure 2A). Withers height was affected by the experimental group and age but not by sex. It was greater in the AI group than in both in vitro counterparts (AI vs. C-IVP: 7.77 ± 1.76 cm; AI vs. RF-IVP: 8.56 ± 2.02 cm; *p* = 0.0008, Figure 2B). Additionally, the thoracic circumference was influenced by the experimental group and age but not by sex. It was greater in AI than in C-IVP (7.06 ± 2.52 cm; *p* = 0.04, Figure 2C). Body length was shaped by age, experimental group, and age-group interaction but not sex. The age-related increase was 1.2-fold greater in the AI and C-IVP groups than in the RF-IVP (*p* = 0.008, Figure 2D).

### 3.2. Hormone Levels

**Figure 3:**
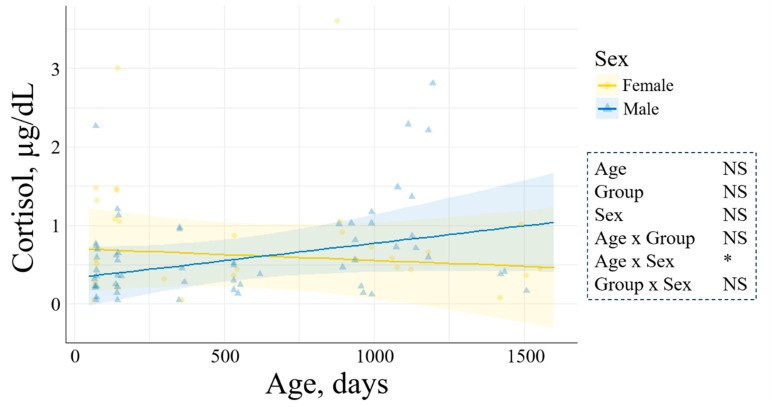
Evolution of cortisol concentrations across age (75 to 1500 days of age) in both females and males. Lines represent the mean cortisol values and haloes the 95% confidence interval. Only in males, the concentration of cortisol significantly increased with age (*p* = 0.039). Only variables with significant differences were represented. Signification codes: NS, not significant; * < 0.05.

**Figure 4:**
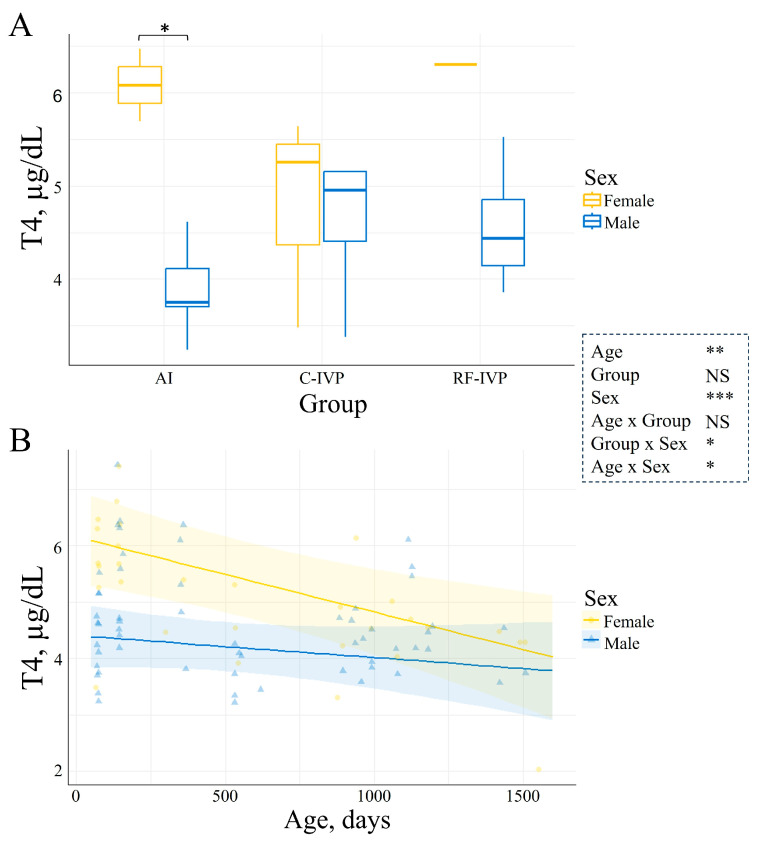
Depiction of the interaction of T4 concentrations with experimental group and age. (**A**) T4 concentration per group and sex at d75. Boxplots represent the median T4 values and the Q1/Q3. In the AI group, T4 concentration was greater in females compared to males (*p* = 0.03). (**B**) Evolution of T4 concentrations across age in females and males. The lines represent the mean T4 values, and the haloes represent the 95% confidence interval. Levels of T4 were reduced with age in both sexes, and this reduction was smaller in males than in females (*p* = 0.025). Only variables with significant differences were represented. Signification codes: NS, not significant; * < 0.05; ** < 0.01; *** < 0.001.

**Figure 5:**
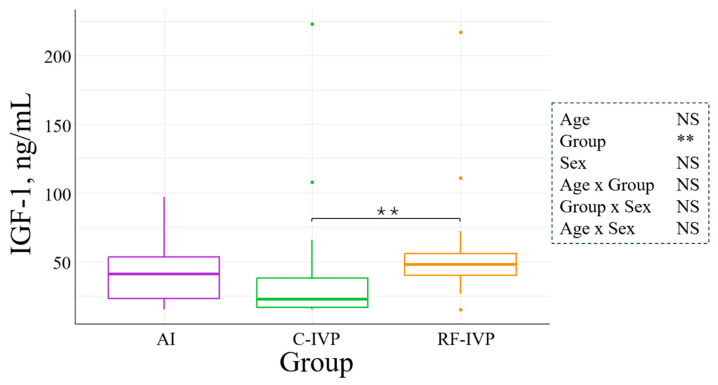
Concentration of IGF-1 at all ages in the different experimental groups. Boxplots represent the median and the Q1/Q3. The IGF concentration was greater in RF-IVP than C-IVP (*p* < 0.0075). Only variables with significant differences were represented. Signification codes: NS, not significant; ** < 0.01.

**Figure 6:**
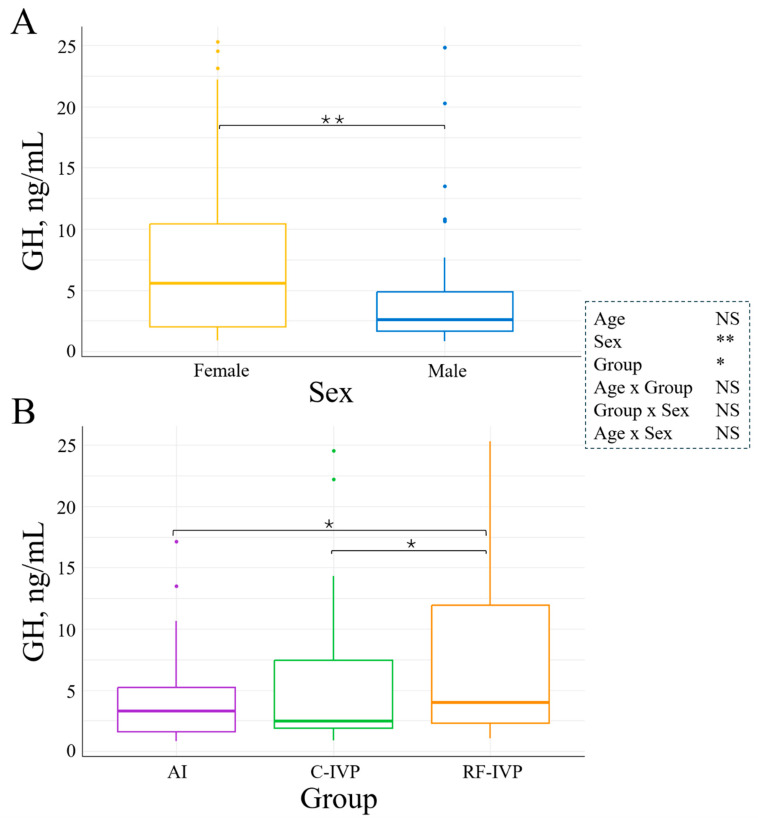
Differences in GH concentrations at all ages between (**A**) males and females and (**B**) experimental groups. Boxplots represent the median GH values and the Q1/Q3. (**A**) Females presented greater GH concentration than males (*p* = 0.004). (**B**) The GH concentration of the RF-IVP group was greater compared to the other two groups (*p* = 0.014). Only variables with significant differences were represented. Signification codes: NS, not significant; * < 0.05; ** < 0.01.

Cortisol was not influenced by either age, sex, or experimental group. However, it was affected by age–sex interaction. Only in males, cortisol levels significantly increased with age, but the increase was only 0.0132 ± 0.0081 $\;\frac{µg}{\mathrm{dL}}/\mathrm{month}$ (*p* = 0.04, Figure 3). Levels of T4 were significantly influenced by age, sex, age–sex, and group–sex interactions. In the AI group, T4 concentration was 1.6-fold higher in females than males at d75 (*p* = 0.0001, Figure 4A). Additionally, T4 was reduced with age in all groups, and this reduction was smaller in males than in females (*p* = 0.03, Figure 4B). The concentration of IGF-1 was not determined by age or sex. However, it was 1.6-fold higher in the RF-IVP than in the C-IVP group (*p* = 0.04), both being similar to AI (Figure 5). Growth hormone was not influenced by age, but it was 4.02 ± 1.52 ng/mL higher in females than in males (*p* = 0.004, Figure 6A) and in the RF-IVP group compared to the AI and the C-IVP groups (4.58 ± 1.85 and 4.18 ± 1.93 ng/mL, respectively; *p* = 0.03, Figure 6B).

### 3.3. Interaction Between Hormone Levels and Growth Parameters

**Figure 7:**
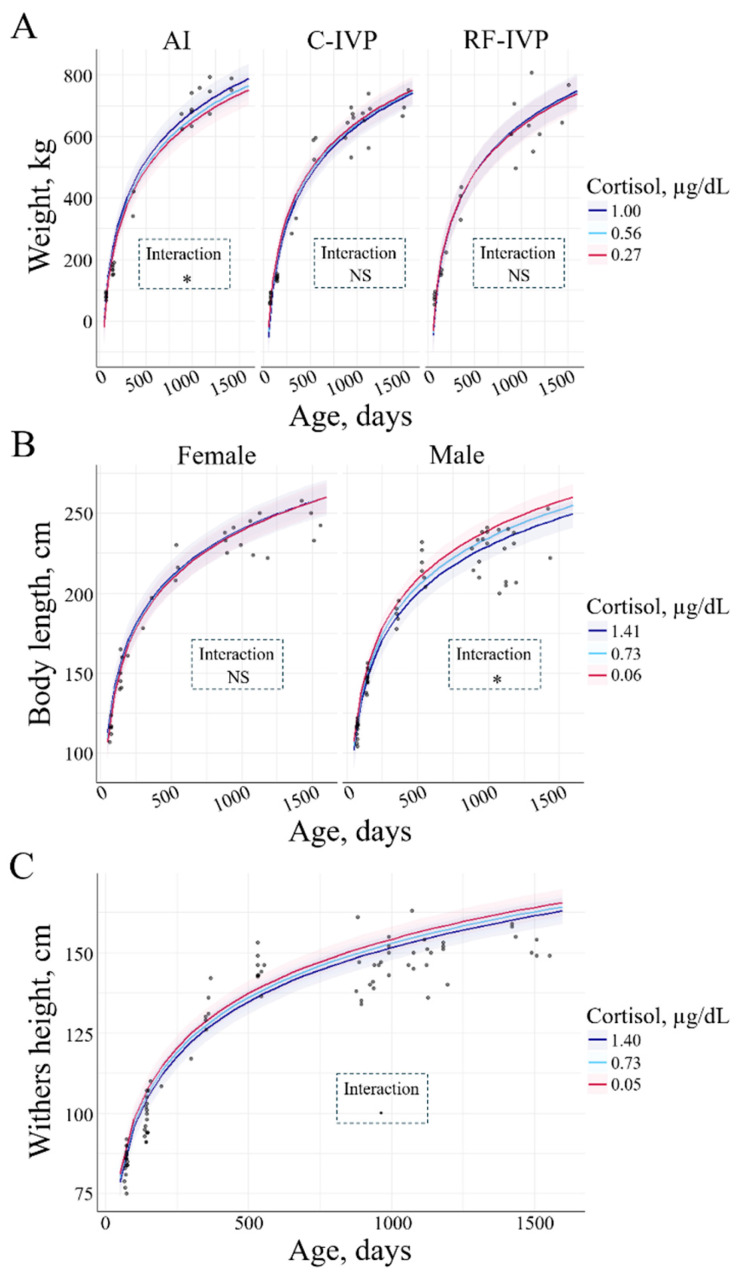
Interaction between cortisol and (**A**) weight, (**B**) body length, and (**C**) withers height across age. The lines represent the mean values of the growth parameters, and the haloes represent the 95% confidence interval. (**A**) There was an interaction between cortisol, weight, and group. In AI, greater cortisol corresponded to greater weight (*p* = 0.043). (**B**) An interaction between cortisol, body length, and sex was observed. In males, greater body length corresponded to lower cortisol (*p* = 0.013). (**C**) A tendency in the relationship between cortisol and withers height was observed. Lower height at withers corresponded to greater cortisol (*p* = 0.06). Only variables with significant differences or a tendency were represented. Signification codes: NS, not significant; • < 0.1; * < 0.05.

A positive interaction was observed between cortisol and weight. In the AI group, higher cortisol was associated with greater weight (34.11 ± 25.78 kg/$\frac{µg}{\mathrm{dL}}$ (*p* = 0.04, Figure 7A). On the contrary, there was a negative interaction between cortisol and body length. In males, the bigger the body length, the lower the cortisol levels (*p* = 0.01, Figure 7B). The interaction between cortisol and withers height was not significant, but a tendency was observed. Higher withers were associated with lower cortisol concentration (*p* = 0.06, Figure 7C). There was no interaction between cortisol and thoracic circumference.

**Figure 8:**
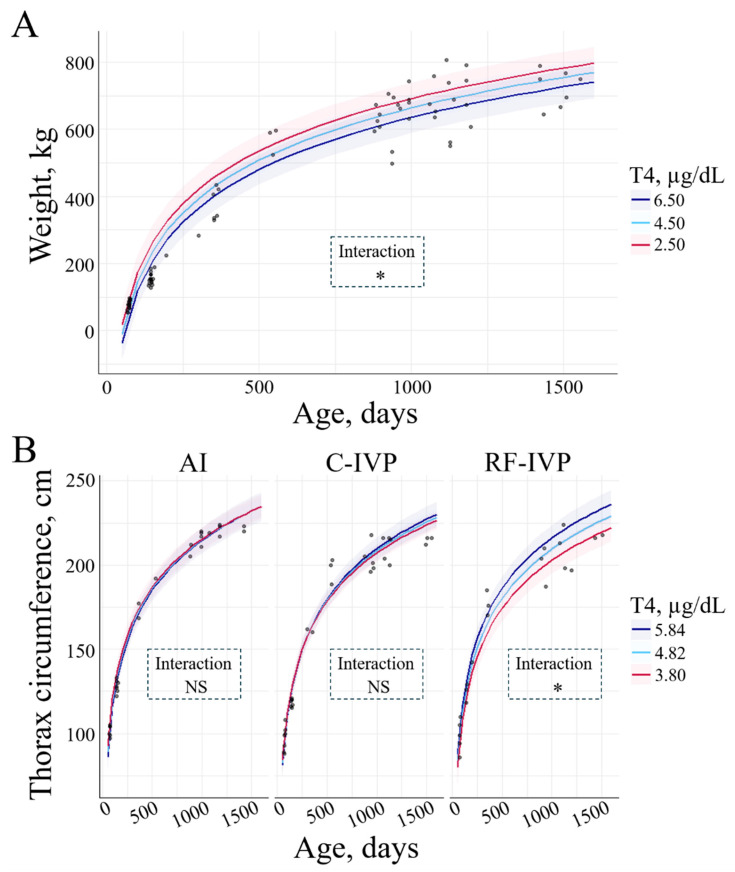
Interaction between T4 and the growth parameters studied across age. Only variables with significant differences were depicted. The lines represent the mean value of the growth parameters, and the haloes represent the 95% confidence interval. (**A**) An interaction between T4 and weight was observed. Greater T4 corresponded to greater weight (*p* = 0.025). (**B**) There was an interaction between T4, thorax circumference, and group. In RF-IVP, the greater the T4, the greater the thorax circumference (*p* = 0.016). Signification codes: NS, not significant; * < 0.05.

Thyroxine and weight showed a negative interaction. Higher T4 levels were associated with lower weight (−13.26 ± 6.49 kg/$\frac{\mu g}{\mathrm{dL}}$; *p* = 0.03, Figure 8A). However, T4 concentration was positively associated with thoracic circumference. In the RF-IVP group, the thoracic circumference was greater, the higher the T4 (5.06 ± 2.67 cm/$\frac{\mu g}{\mathrm{dL}}$; *p* = 0.046, Figure 8B). There was neither an interaction between T4 and withers height nor between T4 and body length.

**Figure 9:**
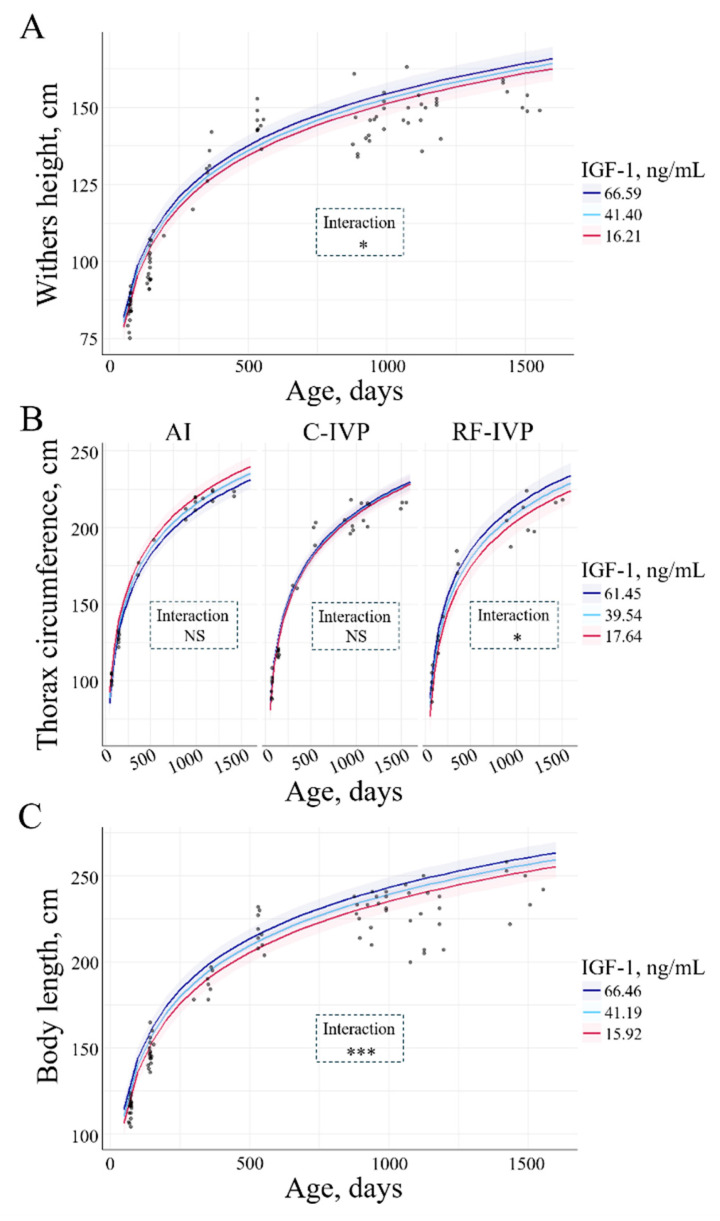
Interaction between IGF-1 and (**A**) withers height, (**B**) thorax circumference, and (**C**) body length across age. Only variables with significant differences were depicted. The lines represent the mean values of the growth parameters, and the haloes represent the 95% confidence interval. (**A**) An interaction between IGF-1 and height at withers was observed. Greater IGF-1 corresponded to greater withers height (*p* = 0.014). (**B**) There was an interaction between IGF-1, thorax circumference, and group. In RF-IVP, the greater the IGF-1, the greater the thorax circumference (*p* = 0.033). (**C**) An interaction between IGF-1 and body length was observed. The greater the IGF-1, the greater the body length (*p* = 0.0002). Signification codes: NS, not significant; * < 0.05; *** < 0.001.

There was no interaction between IGF-1 and weight. Still, there was a positive interaction between IGF-1 and the rest of the growth parameters studied. Higher IGF-1 concentrations were associated with greater height at withers (0.078 ± 0.029 cm/$\frac{\mathrm{ng}}{\mathrm{mL}}$; *p* = 0.01, Figure 9A). In the RF-IVP group, the thoracic circumference was bigger, the greater the IGF-1 (0.28 ± 0.12 cm/$\frac{\mathrm{ng}}{\mathrm{mL}}$; *p* = 0.003, Figure 9B). Additionally, the greater the IGF-1, the higher the body length (0.16 ± 0.04 cm/$\frac{\mathrm{ng}}{\mathrm{mL}};$ *p* = 0.0002, Figure 9C).

**Figure 10:**
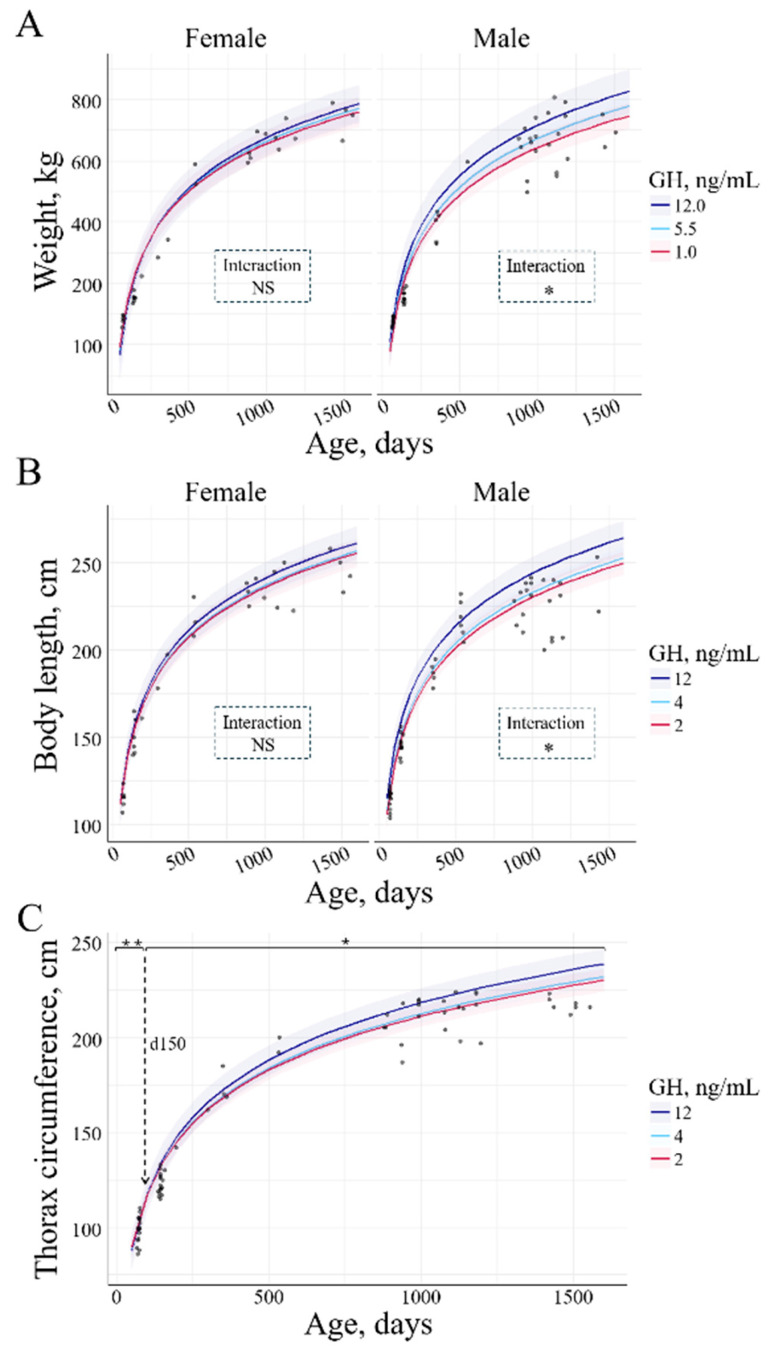
Interaction between GH and (**A**) weight, (**B**) body length, and (**C**) thorax circumference across age. Only variables with significant differences were depicted. The lines represent the mean values of the growth parameters, and the haloes represent the 95% confidence interval. (**A**) There was an interaction between GH, weight, and sex. Only in males, the greater the GH, the greater the weight (*p* = 0.048). (**B**) An interaction between GH, body length, and sex was observed. In males, the greater the GH, the greater the body length (*p* = 0.044). (**C**) An interaction between GH and thorax circumference was observed. There was a negative interaction between thorax circumference and GH until d150 (*p* = 0.0036) and a positive interaction from d150 until d1500 in all the experimental groups (*p* = 0.02). Signification codes: NS, not significant; * < 0.05; ** < 0.01.

A positive interaction of GH with weight and body length was observed in males. Higher GH concentrations were related to greater weight (4.65 ± 2.01 kg/$\frac{\mathrm{ng}}{\mathrm{mL}}$; *p* = 0.03, Figure 10A). Also, higher GH was associated with greater body length (0.93 ± 0.29 cm/$\frac{\mathrm{ng}}{\mathrm{mL}};$ *p* = 0.04, Figure 10B). Between GH and thoracic circumference, there was a negative interaction until d150 (*p* = 0.009) and a positive interaction from d150 until d1500 (*p* = 0.03) in all the experimental groups (Figure 10C). There was no interaction between GH and withers height.

## 4. Discussion

To the best of our knowledge, this is the inaugural study to examine the relationship between growth parameters and growth-influencing hormones in male and female cattle from both in vivo and in vitro origins, from birth up to four years of age. Notwithstanding the aforementioned limitations of this study, namely, the differences in maternal lineage between the in vivo and in vitro groups, the data collected, and the changes associated with age and sex represent a significant contribution to the field. They establish baseline ranges in hormone concentrations and their interactions with growth in this species.

All growth parameters increased with age, but none were influenced by sex. This is probably due to the small number of animals included in this study and the unbalanced sex number.

Previous studies showed an increased birth weight in some calves produced in vitro compared to their in vivo counterparts, mainly related to cell co-culture and fetal bovine serum supplementation [13,14,15]. Additionally, Rerat et al. reported the weight of IVP calves to be greater than that of AI calves from week 8 to week 16, even though the birth weight was similar in both groups [9]. In our study, it was observed that the birth and adult weight of the animals produced in vitro were not different from their in vivo counterparts, even though they differed genetically on the maternal side. This suggests that the culture media used for both in vitro groups supported normal development.

The differences observed in height at withers, being greater in AI compared to the in vitro groups, were most likely related to maternal genetic differences. As previously mentioned, animals from the AI group were crossbred between Holstein and *Asturiana de los Valles* breeds, while animals from the in vitro groups were crossbred between beef cattle (Limousin/Charolais mixed breeds) and *Asturiana de los Valles*. Holstein cattle are, on average, 20 cm higher than Limousin/Charolais animals. In our study, the differences between groups were between 6 and 10 cm, probably as a result of all animals being sired by the same *Asturiana de los Valles* bull. However, the differences in thoracic circumference and body length between the two in vitro groups could not be explained by the differences in the breeds since they were all from the same crossbreed. Animals derived from AI had greater thoracic circumference than C-IVP animals but not than RF-IVP. On the other hand, AI and C-IVP animals were longer than RF-IVP animals. In a previous study including animals from miscellaneous breeds, IVP calves had greater body length than those derived in vivo [9]. Taken together, these data suggest strong similarities between the in vitro groups and AI for different growth parameters. Further studies with a higher number of animals are needed to confirm and explain these findings.

Cortisol concentrations are broadly used to determine stress levels in cattle, with cortisol being positively correlated with stress [8]. Interestingly, Swali et al. reported that cortisol levels were negatively associated with growth parameters (weight, body length, and thoracic circumference) in cattle between 3 and 6 months of age, suggesting that cortisol was a growth inhibitor in soft tissue and bone [16]. Here, we also found a negative association between cortisol and body length in males and withers height at all ages studied. This negative interaction could be due to the growth-inhibiting effect of cortisol or stress-related. Supporting the stress-related option, we also observed that only in males, cortisol levels increased with age. All the bulls of this study were housed together, and as they grew, space became a bit more limited, thus increasing hierarchy-related and population stress. It is easy to imagine that bigger animals would be at the top of the dominance rank, having, therefore, less stress and, in consequence, less cortisol. However, Adeyemo and Heath did not find a correlation between cortisol and dominance [17]. Another reason for the age-related differences in cortisol increase between males and females could be due to differences in handling. After 1 year of age, bulls were only restrained every 6 to 12 months for physical examination and sampling, while cows were regularly restrained for estrous determination and ovarian evaluation by ultrasonography. Therefore, the cows were at ease with handling, being less stressed at sampling. We also observed a positive interaction between cortisol and weight in both males and females of the AI group. The relationship between cortisol and weight is not straightforward; it depends on cortisol responsiveness. In sheep, low cortisol responders are associated with increased skeletal muscle thermogenesis, reduced appetite, and increased activity in response to stress, compared to high cortisol responders [18]. It could be hypothesized that animals from the AI group could be high cortisol responders.

The somatotropic axis, which is primarily composed of growth hormone (GH), insulin-like growth factor 1 (IGF-1), and their associated carriers and receptors [7], is the most significant hormonal regulator of metabolism and growth. The primary source of growth hormone is the pituitary gland; however, it is also produced within reproductive cells, where it is released at a more continuous and lower level than pituitary GH [19]. Growth hormone stimulates the production of IGF-1 in the liver, which then regulates the secretion of GH. Additionally, IGF-1 is produced in a GH-independent manner, with its synthesis being regulated by other factors, including gonadotropins and estradiol.

The present study did not reveal an interaction between growth hormone (GH) and insulin-like growth factor 1 (IGF-1). However, it demonstrated that GH levels were consistently greater in females than males across all age groups. This finding aligns with a previous report [20], which attributed this difference to estrogen action.

Furthermore, there is evidence to suggest that GH levels fluctuate in accordance with an individual’s age. In the immediate postnatal period, GH levels decline and remain at a constant level until the onset of puberty, at which point they undergo a significant increase. Following the onset of puberty, these levels decline at a gradual and continuous rate [9,20]. In contrast, no variation in GH levels with age was observed in our study. This may be attributed to the sampling method employed or the relatively low number of samples taken. It is established that the release of GH follows an ultradian rhythm. Despite the fact that samples were taken between 8 and 11 am, only one sample was taken on each occasion, which means that it was not possible to determine the peak amplitude and frequency values for this hormone. Moreover, the dataset comprises measurements at only two time points prior to puberty (d75 and d150), two time points peri-puberty (d360 and d550), and three time points post-puberty (d900, d1100, and d1500). The combination of these two elements is the most probable cause of the absence of age-related variation in GH levels, in contrast with the detected sex-related differences.

It is established that circulating GH plays a pivotal role in regulating postnatal longitudinal growth [7]. Interestingly, we observed that thoracic circumference, which increased with age, was negatively associated with GH until d150 (pre-puberty) and positively associated after. This suggests that the growth of the thoracic circumference before puberty is not controlled by GH, while after, it becomes GH-driven. Supporting this assumption, a similar GH-growth pattern was observed by Swali et al. in cattle from 3 to 9 months of age. Thoracic circumference was only positively correlated with GH from 6 to 9 months of age [16]. Additionally, they reported that GH was negatively correlated to withers height at 3 months and weight at 6 months but positively correlated to the height increase from 3 to 6 months and the weight from 6 to 9 months [16]. In contrast, we observed no interaction between GH and withers height, but there were positive interactions with weight and body length at all ages, but only in males.

Previous studies have reported an age-dependent increase in IGF-1 levels from d7 to d112 [9], from 3 to 15 months of age [21], and from 3 to 20 months of age [5]. Additionally, male calves have been observed to have greater concentrations of IGF-1 than their female counterparts [5]. In contrast, the present study did not observe any variations in IGF-1 levels related to age or sex. Furthermore, the IGF-1 values reported by Kitagawa et al. for animals with normal growth ranged from 100 to 600 ng/mL, while those for dwarf animals fell within the range of 10 to 200 ng/mL [5]. Despite the use of different techniques for determination, the IGF-1 values observed in our study, ranging from 15 to 200 ng/mL, were unexpectedly more closely aligned with those of dwarf animals than with those of normal growth. A positive interaction was observed between IGF-1 and all growth parameters except weight. Previous authors have reported a positive association between IGF-1 and weight at weaning (6 months of age) as well as a positive association between IGF-1 and the remaining growth parameters at this same age [16,21], which serves to reinforce the pivotal role of IGF-1 in body growth in cattle.

Regarding differences in GH and IGF-1 levels between AI and in vitro-produced animals, we observed higher levels of GH in RF-IVP than in both AI and C-IVP and higher levels of IGF-1 in RF-IVP than in C-IVP, but both were similar to AI. A previous study did not report any differences in GH and IGF-1 concentrations between in vitro-produced and AI calves up to d112 [9]. Surprisingly, the two in vitro groups exhibited disparate GH and IGF-1 levels, and these differences could not be attributed to genetic origin. Moreover, these greater levels of GH and IGF-1 of RF-IVP, compared to C-IVP, were not translated into greater growth. RF-IVP animals showed lower body length than the other two groups and shorter height at withers than AI.

Other key hormones involved in the regulation of metabolism and growth are thyroid hormones [7], which may affect feed intake and modify IGF-1 levels through effects on GH secretion or receptor levels [22]. Additionally, T4 has been demonstrated to influence the growth of skeletal muscle in sheep fetuses, modulating the local activity of the somatotropic axis [23]. However, in the present study, no interaction was observed between T4 and GH/IGF-1.

We observed a reduction in T4 with age (from d75 to d1500), and this reduction was more pronounced in females than in males. Conversely, Takahashi et al. showed the highest levels of T4 at d1, followed by a gradual reduction, reaching levels of adult cows already at 4 weeks of age [24]. Medica et al. reported a drop in T4 levels from d10 to d20, a plateau from d30 to d150, and an increase at d180 and d210 [25]. Moreover, they reported no sex effect on T4 [25], while we observed greater T4 levels in females than in males at d75 in AI animals. Rerat et al. observed a decrease in T4 levels during the first week of life, followed by an increase until d112 [9]. All these studies are short-term and share the T4 concentration drop within the first week of life. Instead, here we depicted the long-term pattern of T4 in cattle, in which short-term fluctuations could have been overseen.

The only interactions observed between T4 and growth parameters were a negative association with weight and a positive association with thoracic circumference, but only in RF-IVP. Furthermore, no differences were observed between the AI and IVP groups. In a study measuring T4 levels between d1 and d112, Rerat et al. reported lower concentrations of T4 in IVP than in AI calves at birth, which may have been due to differences in gestational maturation. However, these differences disappeared by day two [9]. Similar results were observed in cloned calves, which had lower T4 levels at birth than AI animals [26]. The first T4 determination included in the present study was measured at d75; therefore, whether there were also differences in d1 between the AI and IVP animals remains unknown.

## 5. Conclusions

In this observational study, we examined for the first time the relationship between growth parameters and hormones influencing growth and metabolism in male and female cattle from in vivo and in vitro origins from birth up to four years of age. Notwithstanding the constraints imposed by the genetic provenance of the animals under examination, it was evident that in vitro embryo production allowed for the growth and development of healthy animals, as evidenced by comparable growth and hormonal parameters between in vivo and in vitro animals. Nevertheless, further investigation involving a larger herd of animals with homogeneous genetics is required to substantiate these findings. The principal contribution of this study is the collation of novel data on the concentrations of hormones that influence growth in cattle at various stages of life.

## Data Availability

The dataset is available upon request from the authors.

## Supplementary Materials

The following supporting information can be downloaded at https://www.mdpi.com/article/10.3390/ani15050631/s1, Table S1. Details of the experimental group, sex, maternal and paternal genetics and date of birth of each animal included in the study. Table S2. Descriptive data (media, SEM, and maximum and minimum values) of each hormone analyzed per experimental group and age. Values without SEM correspond to single observations. For GH, the 13 values over the detection limit were excluded. These data establish baseline ranges in hormone concentrations in cattle up to 4 years of age, derived from assisted reproductive technologies. These data are foundational, as no comparable information is available for cattle of this age produced through assisted reproductive technologies. Table S3. Descriptive data (media and SEM) of each growth parameter measured per experimental group at representative ages. Values without SEM correspond to single observations. These data establish baseline ranges in growth parameters in cattle up to 4 years of age, derived from assisted reproductive technologies. These data are foundational, as no comparable information is available for cattle of this age produced through assisted reproductive technologies.

## References

[1] Moore S.G., Hasler J.F. (2017). A 100-Year Review: Reproductive Technologies in Dairy Science. J. Dairy Sci..

[2] Lafontaine S., Cue R.I., Sirard M.A. (2023). Gestational and Health Outcomes of Dairy Cows Conceived by Assisted Reproductive Technologies Compared to Artificial Insemination. Theriogenology.

[3] Viana J.H. (2022). Statistics of Embryo Production and Transfer in Domestic Farm Animals. Embryo Technol. Newsl..

[4] Lafontaine S., Labrecque R., Blondin P., Cue R.I., Sirard M.A. (2023). Comparison of Cattle Derived from in Vitro Fertilization, Multiple Ovulation Embryo Transfer, and Artificial Insemination for Milk Production and Fertility Traits. J. Dairy Sci..

[5] Kitagawa H., Kitoh K., Ito T., Ohba Y., Nishii N., Katoh K., Obara Y., Motoi Y., Sasaki Y. (2001). Serum Growth Hormone and Insulin-Like Growth Factor-1 Concentrations in Japanese Black Cattle with Growth Retardation. J. Vet. Med. Sci..

[6] Al-Samerria S., Radovick S. (2021). The Role of Insulin-like Growth Factor-1 (Igf-1) in the Control of Neuroendocrine Regulation of Growth. Cells.

[7] Breier B.H. (1999). Regulation of Protein and Energy Metabolism by the Somatotropic Axis. Domest. Anim. Endocrinol..

[8] Cook N.J. (2012). Review: Minimally Invasive Sampling Media and the Measurement of Corticosteroids as Biomarkers of Stress in Animals. Can. J. Anim. Sci..

[9] Rérat M., Zbinden Y., Saner R., Hammon H., Blum J.W. (2005). In Vitro Embryo Production: Growth Performance, Feed Efficiency, and Hematological, Metabolic, and Endocrine Status in Calves. J. Dairy Sci..

[10] Lopes J.S., Soriano-Úbeda C., París-Oller E., Navarro-Serna S., Canha-Gouveia A., Sarrias-Gil L., Cerón J.J., Coy P. (2022). Year-Long Phenotypical Study of Calves Derived From Different Assisted-Reproduction Technologies. Front. Vet. Sci..

[11] Lopes J.S., Alcázar-Triviño E., Soriano-úbeda C., Hamdi M., Cánovas S., Rizos D., Coy P. (2020). Reproductive Outcomes and Endocrine Profile in Artificially Inseminated versus Embryo Transferred Cows. Animals.

[12] Souza A.H., Ayres H., Ferreira R.M., Wiltbank M.C. (2008). A new presynchronization system (Double-Ovsynch) increases fertility at first postpartum timed AI in lactating dairy cows. Theriogenology.

[13] Yang B.S., Im G.S., Park S.J. (2001). Characteristics of Korean Native, Hanwoo, Calves Produced by Transfer of in Vitro Produced Embryos. Anim. Reprod. Sci..

[14] Behboodi E., Anderson G.B., BonDurant R.H., Cargill S.L., Kreuscher B.R., Medrano J.F., Murray J.D. (1995). Birth of Large Calves That Developed from in Vitro-Derived Bovine Embryos. Theriogenology.

[15] Van Wagtendonk-De Leeuw A.M., Aerts B.J.G., Den Daas J.H.G. (1998). Abnormal Offspring Following in Vitro Production of Bovine Preimplantation Embryos: A Field Study. Theriogenology.

[16] Swali A., Cheng Z., Bourne N., Wathes D.C. (2008). Metabolic Traits Affecting Growth Rates of Pre-Pubertal Calves and Their Relationship with Subsequent Survival. Domest. Anim. Endocrinol..

[17] Adeyemo O., Heath E. (1982). Social Behaviour and Adrenal Cortical Activity in Heifers. Appl. Anim. Ethol..

[18] Lee T.K., Lee C., Bischof R., Lambert G.W., Clarke I.J., Henry B.A. (2014). Stress-Induced Behavioral and Metabolic Adaptations Lead to an Obesity-Prone Phenotype in Ewes with Elevated Cortisol Responses. Psychoneuroendocrinology.

[19] Hull K.L., Harvey S. (2014). Growth Hormone and Reproduction: A Review of Endocrine and Autocrine/Paracrine Interactions. Int. J. Endocrinol..

[20] Wajnrajch M.P. (2005). Physiological and Pathological Growth Hormone Secretion. J. Pediatr. Endocrinol. Metab..

[21] Rodríguez-Sánchez J.A., Sanz A., Tamanini C., Casasús I. (2015). Metabolic, Endocrine, and Reproductive Responses of Beef Heifers Submitted to Different Growth Strategies during the Lactation and Rearing Periods. J. Anim. Sci..

[22] Huszenicza G., Kulcsar M., Rudas P. (2002). Clinical Endocrinology of Thyroid Gland Function in Ruminants. Vet. Med..

[23] Forhead A.J., Li J., Gilmour R.S., Dauncey M.J., Fowden A.L. (2002). Thyroid Hormones and the MRNA of the GH Receptor and IGFs in Skeletal Muscle of Fetal Sheep. Am. J. Physiol. Endocrinol. Metab..

[24] Takahashi K., Takahashi E., Ducusin R.J.T., Tanabe S., Uzuka Y., Sarashina T. (2001). Changes in Serum Thyroid Hormone Levels in Newborn Calves as a Diagnostic Index of Endemic Goiter. J. Vet. Med. Sci..

[25] Medica P., Cravana C., Ferlazzo A.M., Fazio E. (2019). Age-Related Functional Changes of Total Thyroid Hormones and Glycosaminoglycans in Growing Calves. Vet. World.

[26] Garry F.B., Adams R., McCann J.P., Odde K.G. (1996). Postnatal Characteristics of Calves Produced by Nuclear Transfer Cloning. Theriogenology.

