# Supplementary material for: Growth Parameters and Growth-Related Hormone Profile in a Herd of Cattle up to 4 Years of Age Derived from Assisted Reproductive Technologies
Source: Animals (Basel). 2025 Feb 21;15(5):631. doi: 10.3390/ani15050631 (PMC11898124; doi:10.3390/ani15050631)
Supplement: Supplementary file 1 [file animals-15-00631-s001.zip › animals-3411780-supplementary.pdf]

Supplementary material

Table S1. Details of the experimental group, sex, maternal and paternal genetics and date of birth of each animal included in the study.

Calf ID	Group	Sex	Genetics	Date of birth
5523	C-IVP	Male	Maternal: Charolais/Limousin<br>Paternal: Asturiana de los Valles	09/08/2018
5234	C-IVP	Male	Maternal: Charolais/Limousin<br>Paternal: Asturiana de los Valles	16/08/2018
5276	C-IVP	Female	Maternal: Charolais/Limousin<br>Paternal: Asturiana de los Valles	01/09/2018
5860	C-IVP	Male	Maternal: Charolais/Limousin<br>Paternal: Asturiana de los Valles	16/10/2018
2852	C-IVP	Female	Maternal: Charolais/Limousin<br>Paternal: Asturiana de los Valles	03/11/2018
8037	C-IVP	Female	Maternal: Charolais/Limousin<br>Paternal: Asturiana de los Valles	23/02/2019
8067	C-IVP	Male	Maternal: Charolais/Limousin<br>Paternal: Asturiana de los Valles	07/03/2019
5861	RF-IVP	Female	Maternal: Charolais/Limousin<br>Paternal: Asturiana de los Valles	17/10/2018
6809	RF-IVP	Male	Maternal: Charolais/Limousin<br>Paternal: Asturiana de los Valles	11/12/2018
6735	RF-IVP	Male	Maternal: Charolais/Limousin<br>Paternal: Asturiana de los Valles	28/12/2018
8066	RF-IVP	Male	Maternal: Charolais/Limousin<br>Paternal: Asturiana de los Valles	06/03/2019
0507	RF-IVP	Male	Maternal: Charolais/Limousin<br>Paternal: Asturiana de los Valles	19/03/2019
6758	AI	Male	Maternal: Holstein<br>Paternal: Asturiana de los Valles	11/01/2019
6759	AI	Male	Maternal: Holstein<br>Paternal: Asturiana de los Valles	11/01/2019
6762	AI	Female	Maternal: Holstein<br>Paternal: Asturiana de los Valles	11/01/2019
6760	AI	Male	Maternal: Holstein<br>Paternal: Asturiana de los Valles	11/01/2019
6761	AI	Male	Maternal: Holstein<br>Paternal: Asturiana de los Valles	11/01/2019
7918	AI	Male	Maternal: Holstein<br>Paternal: Asturiana de los Valles	29/04/2019
7925	AI	Female	Maternal: Holstein<br>Paternal: Asturiana de los Valles	05/05/2019

Table S2. Descriptive data (media, SEM, and maximum and minimum values) of each hormone analyzed per experimental group and age. Values without SEM correspond to single observations. For GH, the 13 values over the detection limit were excluded. These data establish baseline ranges in hormone concentrations in cattle up to 4 years of age, derived from assisted reproductive technologies. These data are foundational, as no comparable information is available for cattle of this age produced through assisted reproductive technologies.

Group	Age (days)	AI		C-IVP		RF-IVP	
		media ± SEM	max - min	media ± SEM	max - min	media ± SEM	max - min
Cortisol (µg/dL)	75	0.6 ± 0.17	1.5 – 0.1	0.5 ± 0.17	1.3 – 0.1	0.7 ± 0.39	2.3 – 0.2
T4 (µg/dL)	75	4.5 ± 0.44	6.5 – 3.2	4.7 ± 0.34	5.6 – 3.4	4.9 ± 0.44	6.3 – 3.9
IGF-1 (ng/mL)	75	32.4 ± 4.69	48.3 – 15.0	28.4 ± 4.95	49.1 – 15.0	53.0 ± 7.30	76.9 – 34.3
GH (ng/mL)	75	3.0 ± 0.79	6.0 – 0.8	3.5 ± 1.84	14.3 – 0.9	6.1 ± 4.70	24.9 – 1.1
Cortisol (µg/dL)	150	0.5 ± 0.17	1.2 – 0.1	1.1 ± 0.36	3.0 – 0.2	0.9 ± 0.29	1.5 – 0.6
T4 (µg/dL)	150	5.5 ± 0.44	7.4 – 4.2	6.1 ± 0.36	7.4 – 4.5	5.7 ± 0.52	6.4 – 4.7
IGF-1 (ng/mL)	150	54.0 ± 11.20	97.1 – 15.0	28.5 ± 6.64	64.9 – 15.0	80.5 ± 25.38	128.0 – 41.3
GH (ng/mL)	150	2.4 ± 0.83	7.2 – 1.0	5.7 ± 2.90	22.2 – 1.3	6.0 ± 2.28	8.3 – 3.7
Cortisol (µg/dL)	360	0.2 ± 0.11	0.3 – 0.1	0.6 ± 0.32	1.0 – 0.3	0.5 ± 0.27	1.0 – 0.1
T4 (µg/dL)	360	4.6 ± 0.79	5.4 – 3.8	4.9 ± 0.43	5.3 – 4.5	5.8 ± 0.48	6.4 – 4.8
IGF-1 (ng/mL)	360	45.4 ± 30.40	75.8 – 15.0	120.9 ± 102.10	223.0 – 18.8	46.7 ± 11.93	67.8 – 26.5
GH (ng/mL)	360	3.9	-	7.6	-	7.5 ± 3.30	10.8 – 4.2
Cortisol (µg/dL)	550	0.4 ± 0.06	0.5 – 0.2	0.5 ± 0.19	0.9 – 0.3	0.1	-
T4 (µg/dL)	550	4.0 ± 0.38	5.3 – 3.2	4.0 ± 0.32	4.5 – 3.5	4.1	-
IGF-1 (ng/mL)	550	55.0 ± 12.09	95.0 – 31.6	55.9 ± 27.51	108.0 – 22.8	111.0	-
GH (ng/mL)	550	9.0 ± 2.31	13.5 – 2.6	5.1 ± 1.50	6.6 – 3.6	10.7	-
Cortisol (µg/dL)	900	0.9 ± 0.16	1.2 – 0.1	0.9 ± 0.54	3.6 – 0.1	0.9 ± 0.06	1.0 – 0.8
T4 (µg/dL)	900	4.4 ± 0.17	4.9 – 3.8	4.2 ± 0.41	6.1 – 3.3	4.6 ± 0.19	4.9 – 4.2
IGF-1 (ng/mL)	900	28.9 ± 5.18	41.4 – 15.0	19.3 ± 1.46	23.1 – 15.0	15.0 ± 10.23	45.7 – 15.0
GH (ng/mL)	900	6.0 ± 2.42	17.2 – 1.4	9.7 ± 5.06	24.6 – 2.5	3.4	-
Cortisol (µg/dL)	1100	1.1 ± 0.39	2.2 – 0.6	0.9 ± 0.21	1.5 – 0.4	1.6 ± 0.56	2.8 – 0.5
T4 (µg/dL)	1100	4.3 ± 0.10	4.5 – 4.2	4.6 ± 0.31	5.5 – 3.7	5.1 ± 0.48	6.1 – 4.0

**IGF-1 (ng/mL)**	1100	38.8 ± 8.52	54.8 – 16.6	21.9 ± 3.85	31.4 – 15.0	41.1 ± 2.34	47.9 – 37.5
**GH (ng/mL)**	1100	3.9 ± 0.93	5.5 – 1.3	4.0 ± 1.84	7.7 – 2.2	12.2 ± 5.53	23.1 – 2.6
**Cortisol (µg/dL)**	1500	0.2 ± 0.15	0.4 – 0.1	0.6 ± 0.25	1.0 – 0.2	0.4 ± 0.03	0.4 – 0.4
**T4 (µg/dL)**	1500	4.0 ± 0.46	4.5 – 3.6	3.4 ± 0.68	4.3 – 2.0	4.4 ± 0.13	4.5 – 4.3
**IGF-1 (ng/mL)**	1500	70.0 ± 16.75	86.7 – 53.2	62.4 ± 1.96	65.7 – 58.9	134.9 ± 82.15	217.0 – 52.7
**GH (ng/mL)**	1500	3.4 ± 1.43	4.9 – 2.0	4.5 ± 2.64	9.8 – 1.3	7.3 ± 5.09	12.3 – 2.2

Table S3. Descriptive data (media and SEM) of each growth parameter measured per experimental group at representative ages. Values without SEM correspond to single observations. These data establish baseline ranges in growth parameters in cattle up to 4 years of age, derived from assisted reproductive technologies. These data are foundational, as no comparable information is available for cattle of this age produced through assisted reproductive technologies.

Group	Age (days)	AI	C-IVP	RF-IVP
		media ± SEM	media ± SEM	media ± SEM
**Weight (kg)**	0	41.80 ± 2.3	41.33 ± 2.4	41.30 ± 3.7
**Withers height (cm)**	0	79.43 ± 0.9	74.80 ± 0.9	75.00 ± 0.6
**Thorax circumference (cm)**	0	84.29 ± 1.4	73.64 ± 7.5	84.40 ± 1.4
**Body length (cm)**	0	103.71 ± 2.9	105.17 ± 2.4	102.40 ± 2.9
**Weight (kg)**	75	82.79 ± 3.7	75.44 ± 5.6	76.76 ± 7.1
**Withers height (cm)**	75	88.57 ± 0.8	82.86 ± 1.7	82.40 ± 1.6
**Thorax circumference (cm)**	75	101.86 ± 1.2	97.00 ± 2.8	98.80 ± 4.2
**Body length (cm)**	75	118.43 ± 1.5	112.00 ± 2.0	114.40 ± 2.5
**Weight (kg)**	150	169.91 ± 5.6	140.60 ± 3.1	149.40 ± 9.0
**Withers height (cm)**	150	104.71 ± 1.4	94.71 ± 1.3	94.25 ± 1.7
**Thorax circumference (cm)**	150	128.00 ± 1.4	118.29 ± 0.9	121.50 ± 3.7
**Body length (cm)**	150	153.86 ± 2.7	141.29 ± 1.3	144.38 ± 3.9
**Weight (kg)**	360	410.00 ± 16.1	399.74 ± 19.8	391.80 ± 17.4
**Withers height (cm)**	360	140.43 ± 1.7	130.21 ± 1.9	129.80 ± 1.7
**Thorax circumference (cm)**	360	180.21 ± 2.6	172.57 ± 3.0	177.20 ± 2.4
**Body length (cm)**	360	196.00 ± 3.6	199.29 ± 3.1	188.80 ± 3.6
**Weight (kg)**	550	-	583.50 ± 21.3	-
**Withers height (cm)**	550	146.70 ± 2.0	144.33 ± 0.9	136.50
**Thorax circumference (cm)**	550	192.00	197.17 ± 4.4	-
**Body length (cm)**	550	220.00 ± 4.3	216.67 ± 7.5	210.00
**Weight (kg)**	750	613.29 ± 23.3	451.50 ± 12.5	540.00 ± 48.0
**Withers height (cm)**	750	148.43 ± 1.5	134.00 ± 1.0	135.67 ± 3.8
**Thorax circumference (cm)**	750	200.07 ± 2.7	179.00 ± 5.0	188.33 ± 7.0

**Body length (cm)**	750	231.00 ± 6.0	218.50 ± 8.5	214.67 ± 11.5
**Weight (kg)**	900	686.00 ± 19.8	614.14 ± 28.1	598.50 ± 42.8
**Withers height (cm)**	900	151.33 ± 2.6	140.00 ± 2.8	138.25 ± 1.4
**Thorax circumference (cm)**	900	214.00 ± 2.3	202.00 ± 3.6	198.25 ± 5.3
**Body length (cm)**	900	233.50 ± 2.0	226.86 ± 5.5	222.25 ± 4.8
**Weight (kg)**	1100	769.40 ± 33.9	663.20 ± 29.0	650.25 ± 55.1
**Withers height (cm)**	1100	154.75 ± 2.8	148.60 ± 1.1	143.75 ± 3.9
**Thorax circumference (cm)**	1100	220.75 ± 1.7	210.20 ± 3.4	208.00 ± 6.5
**Body length (cm)**	1100	232.75 ± 4.1	228.00 ± 10.6	216.50 ± 5.5
**Weight (kg)**	1300	760.00	722.80 ± 41.9	662.33 ± 35.5
**Withers height (cm)**	1300	175.00	147.40 ± 2.6	152.00 ± 2.6
**Thorax circumference (cm)**	1300	232.00	215.40 ± 4.4	215.33 ± 4.6
**Body length (cm)**	1300	254.00	214.80 ± 18.1	230.33 ± 5.8
**Weight (kg)**	1500	769.50 ± 19.5	703.33 ± 24.7	705.50 ± 60.5
**Withers height (cm)**	1500	158.50 ± 0.5	151.00 ± 1.5	152.00 ± 3.0
**Thorax circumference (cm)**	1500	221.50 ± 1.5	214.67 ± 1.3	217.00 ± 1.0
**Body length (cm)**	1500	255.50 ± 2.5	209.00 ± 37.1	227.50 ± 5.5
